# A Case of a 12-cm Huge Hepatocellular Carcinoma Treated Successfully With Two-Stage CyberKnife Stereotactic Body Radiation Therapy

**DOI:** 10.7759/cureus.83094

**Published:** 2025-04-27

**Authors:** Shuichi Nishimura, Atsuya Takeda, Naoko Sanuki, Takatsugu Kawase, Nobuhiro Tsukamoto

**Affiliations:** 1 Department of Radiation Oncology, Saitama City Hospital, Saitama, JPN; 2 Department of Radiology, Keio University School of Medicine, Tokyo, JPN

**Keywords:** cyberknife, huge hepatocellular carcinoma (hcc), real-time tumor tracking, stereotactic body radiotherapy (sbrt), two-stage sbrt, unresectable hcc

## Abstract

Determining the optimal treatment for patients with huge hepatocellular carcinoma (HCC) remains challenging. Although surgical resection is the preferred option, it is feasible only in selected cases. Transarterial chemoembolization is commonly used but often provides insufficient local control. Advanced radiation therapy methods, such as real-time tumor tracking and inverse planning, have enabled the safe delivery of high-dose radiation via stereotactic body radiotherapy (SBRT), achieving excellent local control. However, alternative strategies may be necessary for difficult cases.

We report the case of an 81-year-old male with a 12-cm unresectable HCC who was successfully treated with two-stage CyberKnife SBRT. Due to the tumor size and proximity to the gastrointestinal tract, single-stage SBRT was considered infeasible. The first-stage SBRT (25 Gy in three fractions, maximum dose of 55 Gy) induced tumor shrinkage, allowing for the second-stage SBRT (25 Gy in five fractions, maximum dose of 50 Gy) six months later. The only adverse event was transient radiation-induced fatigue two weeks post-treatment. A significant reduction in tumor volume was achieved, with no recurrence and preserved liver function for 44 months after initial treatment.

This case highlights the potential of two-stage SBRT with real-time tumor tracking as an effective treatment strategy for unresectable huge HCC.

## Introduction

Stereotactic body radiotherapy (SBRT) has demonstrated favorable local control for small hepatocellular carcinoma (HCC). However, as tumor size increases, achieving local control while preserving liver function becomes increasingly difficult [1]. Even with surgical resection, the five-year survival rate is only 16-42% for patients with huge (>10 cm) HCC [2]. In the updated BCLC staging system of 2022, transarterial chemoembolization (TACE) has been introduced as an option for treatment stage migration in early stage (A) patients [3]. However, most studies on TACE report limited local control and a median overall survival (OS) of less than 12 months [4].

Advanced radiation therapy methods, such as real-time tumor tracking and inverse planning, have enabled the delivery of high-dose radiation to tumors while minimizing radiation exposure to the surrounding normal liver tissue. CyberKnife is a specialized stereotactic radiotherapy system that integrates these features and achieves high positional accuracy, allowing for smaller margins compared to conventional linear accelerator (LINAC)-based radiotherapy systems [5,6]. This precision by real-time tumor tracking enables safe and effective dose escalation to the tumor while minimizing and accurately evaluating the dose to adjacent organs at risk (OARs), such as the gastrointestinal tract.

In this report, we present a case of an unresectable huge HCC that was successfully treated with two-stage CyberKnife SBRT.

## Case presentation

An asymptomatic 81-year-old male was found to have elevated liver enzymes during a routine health checkup. Based on his social history and laboratory findings, alcoholic liver cirrhosis was suspected. The patient's liver function was classified as Child-Pugh class A (5 points). Dynamic contrast-enhanced computed tomography (CT) revealed a 12-cm tumor in the right hepatic lobe, with an enhancement pattern suggestive of HCC. No evident portal vein tumor thrombus or major vascular invasion was observed. Additionally, blood tests showed elevated tumor markers, with a serum alpha-fetoprotein (AFP) level of 100 ng/mL and a protein induced by vitamin K absence-II (PIVKA-II) level of 938 mAU/mL, resulting in a diagnosis of HCC.

At a multidisciplinary HCC conference, surgical resection was not feasible, and the effectiveness of TACE was considered limited due to the involvement of extrahepatic collateral arteries supplying the tumor. Although proton beam therapy was proposed at another institution, the patient declined due to its high cost, as it was not yet covered by Japan's National Health Insurance at that time. Given the tumor size and its proximity to the duodenum, definitive SBRT was deemed challenging. Therefore, a two-stage SBRT approach was planned.

SBRT was performed using the CyberKnife M6 system (Accuray Inc., Sunnyvale, CA), with the Synchrony® real-time tumor tracking system, based on the fiducial gold marker implanted near the tumor. For treatment planning, 4D-CT imaging was performed to delineate the gross tumor volume (GTV) and internal target volume (ITV), as slight respiratory motion-induced displacement was observed between the fiducial marker and the tumor. A 3-mm isotropic margin was added to the ITV to define the planning target volume (PTV). The liver, colon, and duodenum were delineated as OARs.

**Figure 1:**
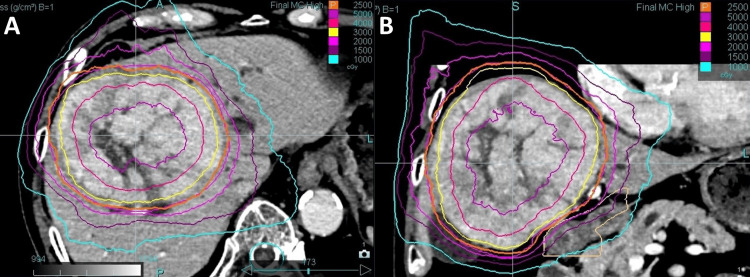
Dose distribution in first-stage SBRT Contrast-enhanced CT showing dose distribution for the first-stage SBRT in the (A) axial and (B) coronal views. A total dose of 25 Gy was delivered in three fractions. Isodose lines, from outermost to innermost, represent 10 Gy, 15 Gy, 20 Gy, 25 Gy, 30 Gy, 40 Gy, and 50 Gy, respectively. SBRT, stereotactic body radiotherapy

In the first-stage SBRT, 25 Gy in three fractions was prescribed to the periphery of the PTV, corresponding to the 45% isodose line of the central dose (Figure 1), primarily to avoid complications involving the duodenum. In addition, a high dose was administered around the center of the tumor to expect tumor volume reduction. The tumor volume was 623 mL, and the liver volume excluding the tumor volume was 1,084 mL. The liver V20Gy was 17.2%, the maximum dose to the duodenum was 21.3 Gy, and the maximum dose to the colon was 23.2 Gy (Table 1).

**Table 1:** The doses in the targets and organs at risk in the ﬁrst-stage and second-stage SBRT BED, biologically effective dose; GTV, gross tumor volume; PTV, planning target volume; SBRT, stereotactic body radiotherapy

Dosimetric parameters	First-stage SBRT	Second-stage SBRT	Cumulative dose
GTV D50 (Gy/BED10)	39.8/92.6	36.6/63.4	76.4/156
GTV D95 (Gy/BED10)	28.0/54.1	27.9/43.5	55.9/97.6
PTV D50 (Gy/BED10)	37.4/84.0	34.7/58.8	72.1/142.8
PTV D95 (Gy/BED10)	25.5/47.2	25.7/38.9	51.2/86.1
Liver volume (mL)	1084	981	
Mean liver dose (Gy/BED3)	12.2/28.7	10.8/18.6	
V20Gy of the liver (%)	17.2	10.7	
Dmax of the duodenum (Gy/BED3)	21.3/71.7	14.3/27.9	35.7/99.6
Dmax of the colon (Gy/BED3)	23.2/83.0	15.0/30	38.2/113

**Figure 2:**
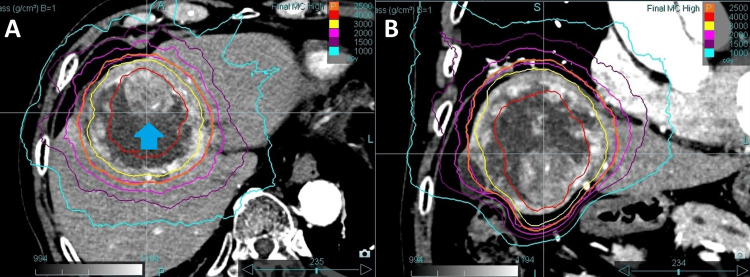
Dose distribution in second-stage SBRT Contrast-enhanced CT showing dose distribution for the second-stage SBRT in (A) axial and (B) coronal views. A total dose of 25 Gy was delivered in five fractions. Isodose lines, from outermost to innermost, represent 10 Gy, 15 Gy, 20 Gy, 25 Gy, 30 Gy, and 40 Gy, respectively. The arrow indicates the recurrently enlarged solid tumor component. SBRT, stereotactic body radiotherapy

Six months after the first-stage SBRT, the tumor volume had decreased to 280 mL; however, regrowth of viable tumor was observed within the necrotic area (Figure 2). Since tumor shrinkage had been achieved by the first-stage SBRT, the increased distance from the adjacent gastrointestinal tract was considered sufficient to safely deliver the second-stage SBRT.

In this second-stage SBRT, 25 Gy in five fractions was prescribed to the PTV periphery, corresponding to the 50% isodose line of the central dose (Figure 2), based on a report indicating that a cumulative maximum dose of BED₃ = 100 Gy_3_ to the gastrointestinal tract is considered safe for re-irradiation [7]. The liver V20Gy was 10.7%, the maximum dose to the duodenum was 14.3 Gy, and the maximum dose to the colon was 15.0 Gy (Table 1). The cumulative dose to the OARs is also shown in Table 1. However, the evaluation of the cumulative liver dose was difficult due to the significant difference in tumor size between the first-stage and second-stage SBRT. In this case, based on previous reports, we concluded that the second-stage SBRT could be safely performed because the patient did not develop radiation-induced liver disease after the first-stage SBRT, maintained good liver function throughout the treatment course, and had a liver volume greater than 700 mL that received less than 15 Gy in both stages [8,9].

**Figure 3:**
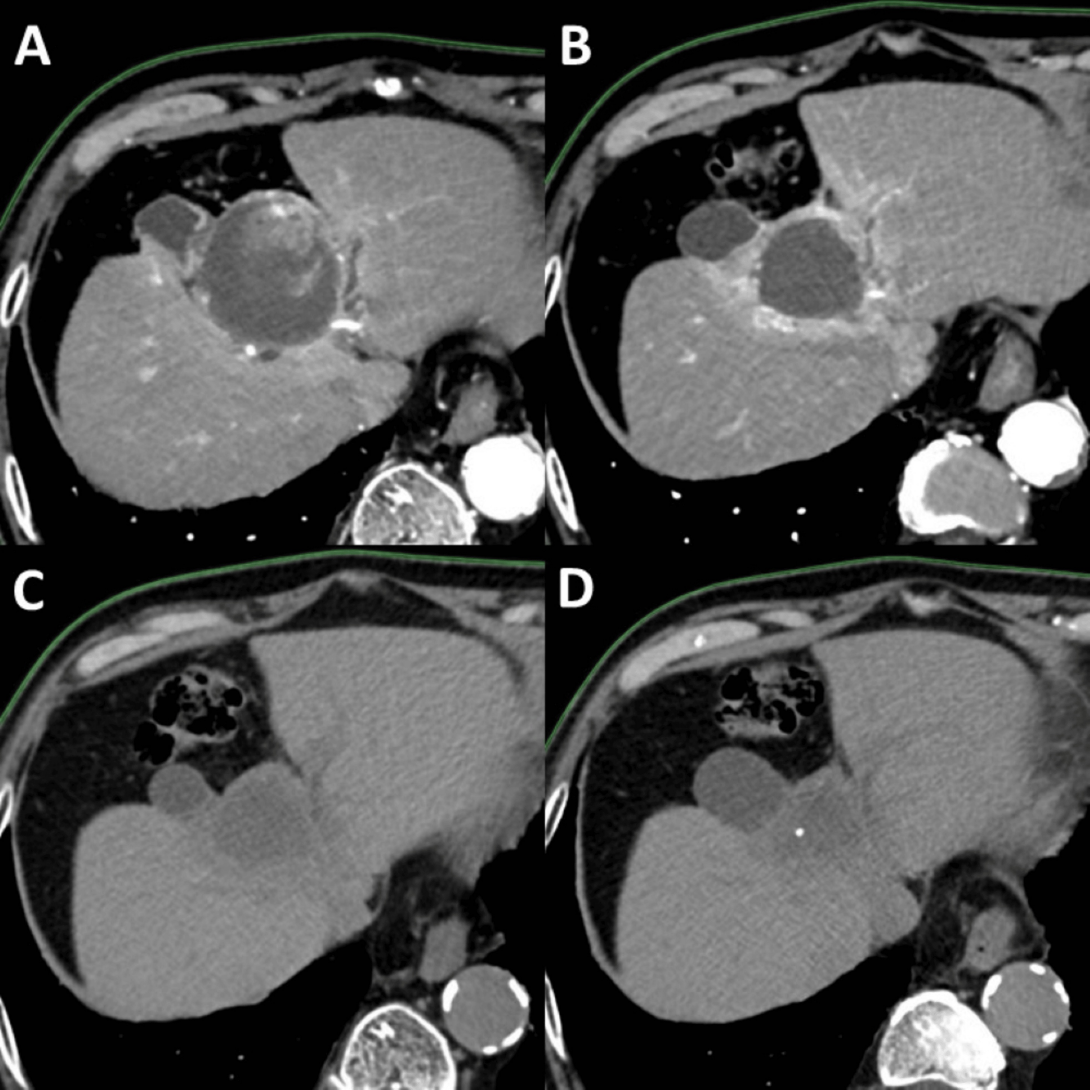
Follow-up CT scans after SBRT The solid tumor component decreased in size at 11 months after the first-stage SBRT (A) and had disappeared by 21 months (B). A complete response was maintained at 32 months (C) and 44 months (D) following the first-stage SBRT. SBRT, stereotactic body radiotherapy

Initially, contrast-enhanced CT was used for surveillance; however, due to the progression of chronic kidney disease, follow-up was subsequently conducted using non-contrast CT and contrast-enhanced ultrasonography. At 44 months after the first-stage SBRT, no evidence of local recurrence or distant metastasis was observed (Figure 3). AFP and PIVKA-II decreased and have remained within normal limits since six months after the first-stage SBRT. Liver function has been maintained at Child-Pugh class A (5 points) from the initial treatment to the present. The only noted adverse event was transient radiation-induced fatigue lasting approximately two weeks after the first-stage SBRT. No additional treatments for HCC other than SBRT were administered.

## Discussion

SBRT has demonstrated high efficacy for HCC and is applicable to cases with vascular invasion or portal vein tumor thrombus [10,11]. In addition, SBRT allows for the adjustment of treatment intensity based on the balance between tumor size, liver volume, and dose constraints of adjacent OARs. Consequently, even for patients with limited treatment options, it is possible to develop a treatment strategy within a relatively safe range. Radiation oncologists evaluate dose constraints for the OARs and aim to deliver the highest possible dose to the target volume while adhering to these limits. Nevertheless, in patients with large tumors located near the gastrointestinal tract, designing a meaningful SBRT plan that delivers an adequate radiation dose remains challenging.

Proton beam therapy is an established treatment for huge HCC and has a physical advantage in reducing radiation dose to surrounding normal tissues [12]. However, when proton beam therapy is not indicated, SBRT using a LINAC serves as a promising alternative. In particular, real-time tumor tracking allows for minimizing the irradiated volume while increasing dose intensity, which is expected to improve treatment outcomes. The CyberKnife system can achieve an overall targeting accuracy of ≤0.95 mm for targets affected by respiratory motion, potentially reducing or eliminating the internal margin required in the conventional LINAC [13].

In this report, we present a case in which a huge HCC was successfully controlled with SBRT alone. There are reports of SBRT being used combined with treatments like TACE for huge HCC [14,15]; however, reports of successful tumor control with SBRT alone are still rare. In the present case, the key to treatment success was the use of a high-precision irradiation technique, which enabled a lower peripheral dose to the PTV to minimize adverse events, while simultaneously maximizing the internal tumor dose to enhance treatment intensity.

Furthermore, a two-stage SBRT approach was employed in this case. This method has been reported as an effective treatment strategy for large brain metastases, achieving a moderate tumor volume reduction one month after the first-stage stereotactic radiosurgery (SRS), followed by the second-stage SRS [16]. In this case, first-stage SBRT was performed not with the goal of immediate tumor control but to reduce tumor size as much as possible because adjacent gastrointestinal tracts became dose-limiting factors, preventing the delivery of adequate enough radiation dose to control the tumor. Second-stage SBRT was administered after achieving sufficient separation between the tumor and adjacent gastrointestinal tracts, allowing for a higher radiation dose to the tumor. To enhance tumor volume reduction, the peripheral radiation dose was set relatively low while the central dose was maximized. Previous studies of SBRT for lung cancer have revealed that increasing the central dose contributes to improved local control [17], supporting this approach. This strategy is consistent with partially ablative body radiotherapy (PABR), a form of spatially fractionated radiation therapy. In PABR, a high ablative dose is delivered to the tumor core while a lower dose is maintained at the tumor margins. This technique has been studied recently and has been suggested to achieve significant tumor shrinkage while minimizing toxicity, particularly in bulky tumors [18-20].

In conclusion, we report a case of successful tumor control in a patient with huge unresectable HCC treated with two-stage SBRT using real-time tumor tracking irradiation. Our findings suggest that SBRT may serve as a promising alternative treatment for patients with unresectable huge HCC.

## Conclusions

This case demonstrates the effectiveness of two-stage CyberKnife SBRT in treating unresectable huge HCC. By using the Synchrony® real-time tumor tracking system, central tumor dose escalation, and tumor volume reduction after the first-stage SBRT, sustained local control was achieved without liver or gastrointestinal dysfunction. This approach enabled the safe delivery of higher radiation doses while minimizing toxicity to adjacent OARs. Our findings suggest that two-stage SBRT could be a promising alternative for patients with unresectable huge HCC, providing a potential therapeutic option when other treatments are limited.
